# Health Boards and Quarantines

**Published:** 1905-10

**Authors:** 


### EDITORIAL NOTES AND COMMENTS.

The Business office of THE JOURNAL-RECORD is No. 1007-1008 Century Building.

The Editorial office is 318-319-320 The Prudential.

Address all Business communications to Dr. M. B. Hutchins, Mgr.

Make remittances payable to THE ATLANTA JOURNAL-RECORD OF MEDICINE.

On matters pertaining to the Editorial and Original Communications address Dr. Bernard Wolff, Atlanta.

Reprints of original articles will be furnished at cost price. Requests for the same should always be made *on the manuscript.*

We will present, post-paid, on request, to each contributor of an original article, twenty (20) marked copies of THE JOURNAL-RECORD containing such article.

### HEALTH BOARDS AND QUARANTINES.

THE incident of a case of yellow fever developing in Atlanta has been the occasion of testing the scope and authority of the State Board of Health as measured with that of the municipal board.

A brief history of the incidents connected with the clash between the two bodies may be of interest, although the publicity given them through the newspapers may make the narrative stale reading.

On Friday, August 31, a man who had recently come from Pensacola, and who was detained at the temporary hospital at the old dumping-grounds, in the western part of the city, was officially declared to be suffering from yellow fever. This intelligence was promptly transmitted by the health officer, Dr. J. P. Kennedy, to the mayor, who in pursuance of the premeditated policy of publicity of the Atlanta Board of Health, telephoned a report of the case to the newspapers.

A reporter connected with one of the papers then communicated with Dr. H. F. Harris, Secretary of the State Board of Health, and ascertained from him that his board had not been informed of the existence of a case of yellow fever in Atlanta. The State Board of Health was then in session and was informed of the occurrence by Dr. Harris, and as a result officially censured the Atlanta Board of Health, through its officials, for failure to promptly report the matter to the State body. This note of censure was followed by an order to the city of Atlanta to establish a quarantine against the infected points. At a meeting of the city board on Sunday, September 2, the censure of its officials was declared unwarranted, and a motion to defer action and quarantine prevailed by vote of 5 to 3.

Dr. Westmoreland appeared at this meeting and declared that in the event Atlanta did not institute quarantine, that the health certificates then being issued by the city would be invalidated by the State board.

Following this, it was announced that the State board would place inspectors on all incoming trains from the infected territories, who were to enforce quarantine with all the pains and penalties attached to willful infractions of the regulations.

A dramatic incident was the alleged barricade of a coach containing refugees against Dr. Harris, who demanded admittance for the purposes of inspection, and the reception at the terminal station of the refugees by the mayor and a squad of policemen, offering them the freedom of the city.

Then, on the somewhat fallacious principle of the greater including the less, the State board declared a quarantine of the whole State against points designated as infected and made the announcement that the regulations of the quarantine would be carried out *vi et armis*. Presumably of the nature of a reprisal was the apprehension on a train, near the suburbs of East Point, of an inspector from the City Board of Health by a State Board of

Health inspector, and the subsequent enlargement of the *corpus delicti* when within the city limits.

This brings the affair, which presents some of the features of a comic opera, up to the time of this writing. How the muddle will be settled, and the jarring sections join hands and dance before the footlights at the final fall of the curtain, one may not forecast.

There are many in the profession and a few out of it who are familiar with the struggle which the State board has made for establishment and recognition, and are fully aware of the unselfish motives which actuate the officials in their endeavor to protect what they believe to be the welfare of the people. But there are not a few who are inclined to question the advisability and expediency of the methods pursued, especially just at the time when the State board is hanging, so to speak, by the eyelids, in the matter of serious recognition and support on the part of the people.

Whatever the outcome be, undoubtedly some good must accrue in the way of establishing the legal status of the State and municipal boards and the formation of a precedent for future guidance and for harmonious co-operative action.

The stumbling-block in the way of agreement will ever lie in the contending views as to the infectibility of Atlanta, and this is an obstacle that can be removed only by the revelations of science and exact knowledge of the means and channels of disease transmission.

---

CENSUS takers have found, says a contemporary, the largest family in Wisconsin at Littel Christie. Mr. and Mrs. Anton Verkullen are parents of twenty-seven children, all but three of whom are living, and most of them are still under the parental roof. Verkullen is fifty-three years old, and his wife is one year his senior. They were married June 1, 1875. In the family are three sets of twins. Five of the children are married, and the oldest is the father of four children.

